# A novel small molecular NLRP3 inflammasome inhibitor alleviates neuroinflammatory response following traumatic brain injury

**DOI:** 10.1186/s12974-019-1471-y

**Published:** 2019-04-11

**Authors:** Ram Kuwar, Andrew Rolfe, Long Di, Hongyu Xu, Liu He, Yuqi Jiang, Shijun Zhang, Dong Sun

**Affiliations:** 1 Department of Anatomy and Neurobiology, School of Medicine, Medical College of Virginia Campus, Virginia Commonwealth University, Richmond, VA 23298-0709 USA; 2 Department of Medicinal Chemistry, School of Pharmacy, Virginia Commonwealth University, Richmond, VA 23298-0709 USA

**Keywords:** Traumatic brain injury, Neuroinflammation, NLRP3, Inflammasome, Cytokines

## Abstract

**Background:**

Neuroinflammation is an essential player in many neurological diseases including traumatic brain injury (TBI). Recent studies have identified that inflammasome complexes are responsible for inflammatory responses in many pathological conditions. Inflammasomes are intracellular multiprotein complexes which regulate the innate immune response, activation of caspase-1, production of pro-inflammatory cytokines IL-1β and IL-18, and induction of cell death (pyroptosis). Among inflammasome family members, the nucleotide-binding domain leucine-rich repeats family protein 3 (NLRP3) is the most extensively studied and its activation is induced following TBI. As a novel target, drug development targeting the formation and activation of NLRP3 inflammasome is a prospective therapy for TBI. We have recently developed a small molecule JC124 with specificity on NLRP3 inflammasome. In this study, we explored the therapeutic value of JC124 for TBI treatment.

**Methods:**

Adult male Sprague-Dawley rats were subjected to a moderate cortical impact injury. Following TBI, animals received 4 doses of JC124 treatment with the first dose starting at 30 min, the second dose at 6 h after TBI, the third and fourth doses at 24 or 30 h following TBI, respectively. Animals were sacrificed at 2 days post-injury. Brain tissues were processed either for ELISA and western blotting analysis for inflammatory response, or for histological examination to assess degenerative neurons, acute inflammatory cell response and lesion volume.

**Results:**

We found that post-injury treatment with JC124 significantly decreased the number of injury-induced degenerating neurons, inflammatory cell response in the injured brain, and cortical lesion volume. Injured animals treated with JC124 also had significantly reduced protein expression levels of NLRP3, ASC, IL-1 beta, TNFα, iNOS, and caspase-1.

**Conclusion:**

Our data suggest that our novel NLRP3 inhibitor has a specific anti-inflammatory effect to protect the injured brain following TBI.

## Introduction

Traumatic brain injury (TBI) is a major health problem worldwide. Currently, there is no effective treatment. Following TBI, the primary injury induces irreversible and untreatable brain damage. The subsequent secondary injury plays a profound role in the evolution of injury and clinical prognosis. Thus, preventing/treating the additional tissue damage caused by secondary brain insults is the major focus of therapies for TBI. Among secondary injuries following TBI, neuroinflammation is a prominent event that significantly exacerbates brain tissue damage causing functional deficits. To date, abundant studies have shown that targeting neuroinflammation is a promising strategy for TBI treatment.

Inflammation is mediated by inflammatory cells and inflammatory cytokines/chemokines they released. Among TBI-induced pro-inflammatory cytokines, interleukin-1β (IL-1β) plays a pivotal role in triggering TBI-induced inflammatory cascade [1]. Another cytokine, interleukin-18 (IL-18) has been known as a potent inflammatory mediator that initiates/amplifies many inflammatory processes [2]. The brain is particularly sensitive to IL-1β and IL-18 signaling because multiple neural cell types in the CNS express receptors for these cytokines [3, 4]. Recent studies have found that release of IL-1β and IL-18 is mediated by inflammasomes [5]. Inflammasomes are intracellular multiprotein complexes which regulate the innate immune response, activation of caspase-1, production of pro-inflammatory cytokines such as IL-1β and IL-18, and induction of cell death (pyroptosis) [6, 7]. The inflammasomes share a similar structure and are typically formed by a cytosolic pattern-recognition receptor, an adaptor protein, and an effector component (caspase-1) [6]. Among the known inflammasomes, the nucleotide-binding domain leucine-rich repeats family protein 3 (NLRP3) is the most extensively studied and widely implicated regulator of caspase-1 activation, the maturation and production of IL-1β and IL-18 [7]. Activation of NLRP3 is induced by multiple stimuli, including reactive oxygen species, mitochondrial damage, ATP, and potassium ion efflux from injured cells following tissue damage [7]. NLRP3 inflammasome which consists with the NLRP3 scaffold, the apoptotic speck-containing protein (ASC) adaptor and caspase-1, has been reported to associate with neuroinflammation in Alzheimer’s disease (AD) [8–10], Huntington’s disease [11], and pneumococcal meningitis [12]. Increased formation of NLRP3 inflammasome complex in the injured cerebral cortex has been reported following TBI [13], and inflammasome-induced cell destruction is considered to be responsible for post-TBI amplification of the initial tissue damage [14]. As a novel target in neuroinflammation signaling pathway, drug development targeting the formation and activation of inflammasomes is a prospective therapy for TBI.

In this study, we explored the potential of a novel NLRP3 inflammasome inhibitor for TBI therapy. We have recently developed a specific NLRP3 inflammasome inhibitor, JC124, through structural optimization of glyburide, an FDA approved anti-diabetic drug (sulfonylurea) that has been shown to inhibit NLRP3 inflammasome formation [15]. However, the high dose required for glyburide’s *in vivo* NLRP3 inhibition causes lethal hypoglycemia. Through rational design, our novel compound JC124 has shown selective inhibition of NLRP3 inflammasome formation and activation of caspase-1, and reduction of IL-1β both in vitro and in vivo [16]. In a mouse acute myocardial infarction model, JC124 treatment blocked inflammasome formation and reduced myocardial infarct size significantly while exhibited no hypoglycemia effects that clearly demonstrated its target engagement and in vivo activities [17, 18]. Treatment of AD transgenic mice with JC124 also significantly improved multiple AD pathologies including inflammatory responses [19]. In this proposal, we investigated the therapeutic effects of JC124 following TBI in a rat focal contusion injury model. We speculate that NLRP3 inflammasome generated following TBI plays an important role in the progression of brain tissue damage, and targeting NLRP3 inflammasome with our novel compound will have a protective effect.

## Materials and methods

### Animals

A total of 31 male 3–4-months-old Sprague-Dawley rats (Envigo, NJ) weighing approximately 300 g were included in this study. Animals were housed in the animal facility, with a 12-h light/dark cycle, water and food provided ad libitum. All procedures were approved by our Institutional Animal Care and Use Committee.

### Surgical procedures

Animals were subjected to a moderate controlled cortical impact injury (CCI). Briefly, adult rats were anesthetized in a plexiglass chamber with 5% isoflurane, intubated and ventilated with 2% isoflurane in a gas mixture (30% O_2_, 70% N_2_), and fixed on a stereotaxic frame. After a midline incision and skull exposure, a 4.9 mm craniotomy was trephined on the left parietal bone half way between the lambda and bregma sutures. A moderate CCI was induced using an electromagnetic impact device (Leica, Germany) with a 3 mm impactor tip with a velocity of 3.5 m/s, dwell time 0.5 s, and the depth at 2.5 mm. This injury intensity produces a focal cortical contusion without damaging the hippocampus. Sham animals went through the same aesthetical procedures and received skin incision only. After the injury, the skin incision was sutured, 2% lidocaine hydrochloride jelly and antibiotic ointment were applied topically. The animal was returned to a warm cage. Injured animals were subsequently randomized into drug and vehicle treatment groups, and subsequent analysis was done blinded. Animal numbers for each study were determined by past experience and power analysis using SYSTAT software with the power set at 0.80, alpha at 0.05, sigma at 0.97, and mean differences set at 1.95 for a two-way ANOVA. JC124 was administrated i.p. at the dose of 100 mg/kg according to our published study showing the efficacy of JC124 in a mouse acute myocardial infarction model [17], with the first dose given at 30 min post-injury, the second, third, and fourth dose given at 6, 24, and 30 h after TBI, respectively. The treatment time points were selected as TBI induces upregulation of pro-inflammatory cytokines such as IL-1β, IL-6 rapidly within 48 h after injury [20, 21]. Control animals were treated with an equal volume of vehicle solution (10% DMSO in PEG-100).

### Tissue preparation

Animals were sacrificed at 2 days post-injury. The rat was deeply anesthetized with an overdose of isoflurane inhalation, and the blood was drawn with a transcardial puncture. For ELISA and western blotting study, animals were subsequently perfused with 150-ml ice-cold phosphate-buffer saline (PBS) (*N* = 5 for each group). The brains were quickly dissected on ice, ipsi- and contra-lateral cerebral cortex, and hippocampus were dissected separately and homogenized with RIPA buffer (Stock 10X RIPA, EMD Millipore, MA) containing 10% TritonX-100,10% SDS solution, Protease inhibitor, 0.5 M EDTA. Homogenates were centrifuged at 14000 rpm for 25 min, and supernatants were collected and stored at − 80 °C until use. The total protein concentration was determined by BCA method (Pierce, Rockford, IL). For histology, animals were perfused with 150 ml PBS followed by 150 ml 4% paraformaldehyde in PBS (*N* = 4 for sham, *N* = 6 for TBI-vehicle and TBI-JC124 treated groups). The brains were dissected and post-fixed in 4% paraformaldehyde for 48 h at 4 °C and then cut coronally at 60 μm with a vibratome throughout the rostrocaudal extent of the brain. Sections were collected in 24-well plates filled with PBS plus 0.05% sodium azide and stored at 4 °C until use.

### ELISA

The level of pro-inflammatory cytokines IL-1β and IL-18 in the serum and the cortical brain tissue lysate were estimated using ELISA kits (IL-1β Rat ELISA Kit #ab100767, Abcam, MA, USA; IL-18 Rat ELISA kit # KRC2341, Novex by life technologies, USA) following manufacturer’s instructions. Limits of detection for the ELISA kits were IL-1β = 68.6–5000 pg/ml and IL-18 = 15.6–1000 pg/ml. TNFα was measured in the ipsilateral cortical and hippocampal tissue lysates (TNFα Rat ELISA kit #ab100785, Abcam, MA, USA).

### Western blotting

Ipsilateral hippocampal tissue lysate was processed for Western Blotting. For each sample, 20 μg of protein was loaded in each well of 4–12% SDS-PAGE Criterion Gel TGX stain-free gel (Bio-Rad, Hercules, CA, USA). The gel was activated in the UV light using the Chemidoc MP imaging system (Bio-Rad, USA) for 45 s before blotting, and then blotted to PVDF membrane. The transfer of protein was done using Trans-Turbo Blot transfer system (Bio-Rad, USA). The stain-free image of the blot was obtained after transfer for normalization of the blot. The transferred membranes were then blocked for 1 h in 5% milk made in TBST at room temperature and incubated with primary antibodies. The following primary antibodies were used: NLRP3 (1:800; ab214185, Abcam, MA, USA), ASC (1:1000; AL177, AdipoGen, CA, USA), caspase-1 P10 (1:500; sc-56,036, Santa Cruz, CA, USA), iNOS (1:1000; ab3523, Abcam, MA, USA), Arginase-1 (1:1000; sc-20,150, Santa Cruz, CA, USA), and IL-1 beta (1:1000; ab2105, Abcam, MA, USA). After the primary antibody incubation, membranes were thoroughly washed 5 times, 5 min each with 5% milk in the TBST. The membranes were then incubated with the appropriate secondary antibodies for 1 h at room temperature. The secondary antibodies used were horseradish peroxidase-conjugated anti-rabbit, anti-mouse, or anti-goat IgG (1:5000; Cell Signaling, MA, USA). The membranes were then washed 5 times and developed with the Clarity Western ECL Substrate (Bio-Rad, USA) and the chemiluminescent images were captured using the ChemiDoc MP imaging system (Bio-Rad, USA). The analysis of the images was done using the Image Lab 6.0 software (Bio-Rad, USA). The stain-free image of blots was used for the total protein normalization against the chemi-luminescent images. The normalized volume intensity was plotted as densitometry values in the form of histograms as published before [22].

### FJB staining

To specifically assess degenerating neurons following injury, 60-μm-thick coronal brain sections were processed for Fluoro-Jade B (FJB) staining following our published protocol [23]. Briefly, for each brain, 4 sequential coronal sections with 480 μm apart at the level of hippocampus from 2.56 mm to 5 mm of the bregma level were mounted on superfrost plus slides and air dried. The sections were first treated with 1% NaOH in 80% ethanol for 5 min and then were hydrated in graded ethanol and distilled water. They were then incubated in 0.06% potassium permanganate solution for 10 min, followed by a quick rinse and incubation with 0.0004% FJB (Histo-Chem, Inc., Jefferson, AR) plus 0.0004% DAPI (Sigma-Aldrich, St. Louis, MO) solution for 20 min. The slides were then dried, immersed in Citra Solv (Citra Solv, Danbury, CT), and cover-slipped.

### Immunohistochemistry

To assess inflammatory cell response, we used antibodies OX6 and ED1 to stain inflammatory cells. OX6 stains MHC class II antigens expressed on antigen presenting cells including infiltrating macrophages, activated microglia and leukocytes, whereas ED1 stains lysosomes in infiltrating macrophages and activated microglia [24]. For each brain, every eighth section at the level of hippocampus from 2.56 mm to 5 mm of the bregma level were processed for OX6 or ED1 immunostaining following our previously published protocol [25]. Briefly, the sections were washed with PBS and endogenous peroxidase was blocked using 3% H_2_O_2_. Following an overnight serum blocking with 5% normal horse serum in PBS, sections were incubated with mouse anti-OX6 (1:500, Serotec, UK) or mouse anti-ED1 antibody (1:500, Chemicon) in PBST (PBS with 0.4% Triton) plus 5% normal horse serum at 4 °C for 48 h with agitation on shaker. After rinsing with PBST, sections were incubated with biotin-conjugated anti mouse-IgG (1:200, Jackson Laboratory) overnight at 4 °C and then incubated with ABC complex for 2 h at room temperature before visualized with 5.5 diaminobenzidine (DAB). Sections were mounted on glass slides, lightly counterstained with 0.1% cresyl violet, and coverslipped.

### Lesion volume assessment

To measure the cortical lesion volume, 60-μm-thick sequential coronal sections space between 480 μm spanning the entire rostrocaudal extent of the injured cortex were mounted on slides and stained with hematoxylin and eosin (H&E). Cortical lesion size was measured with ImageJ program by outlining the injured brain area. Lesion volume was calculated using the areas, distance between sections, and section thickness.

### Cell quantification

The number of FJB+, OX6+, or ED1+ cells in the ipsilateral cortex and dentate gyrus of the hippocampus was quantified from each section using ImageJ program. For FJB staining, sections containing the hippocampus were examined with an Olympus fluorescent microscope using a × 20 objective lens, and images were captured. For OX6- or ED1-stained sections, an Olympus light microscope was used with a × 20 objective lens, and images were captured. The number of FJB+, OX6+, or ED1+ cells in the ipsilateral cortex and hippocampal dentate gyrus (granular cell layer and hilus regions) was counted separately by a blinded observer with the ImageJ automated counting program. The number of counted cells from four sections per brain was averaged and expressed as the number of cells per square millimeter.

### Statistical analysis

The data generated was analyzed using the GraphPad Prism 7.0 software. A one-way ANOVA followed by Tukey’s post hoc test for the multiple comparison or the Student *t* test was utilized, with *p* value less than 0.05 considered statistically significant. Data are presented as mean ± SEM in all figures.

## Results

### JC124 treatment abolishes TBI-enhanced protein expression of NLRP3 and its adaptor protein ASC in the injury brain

**Fig. 1:**
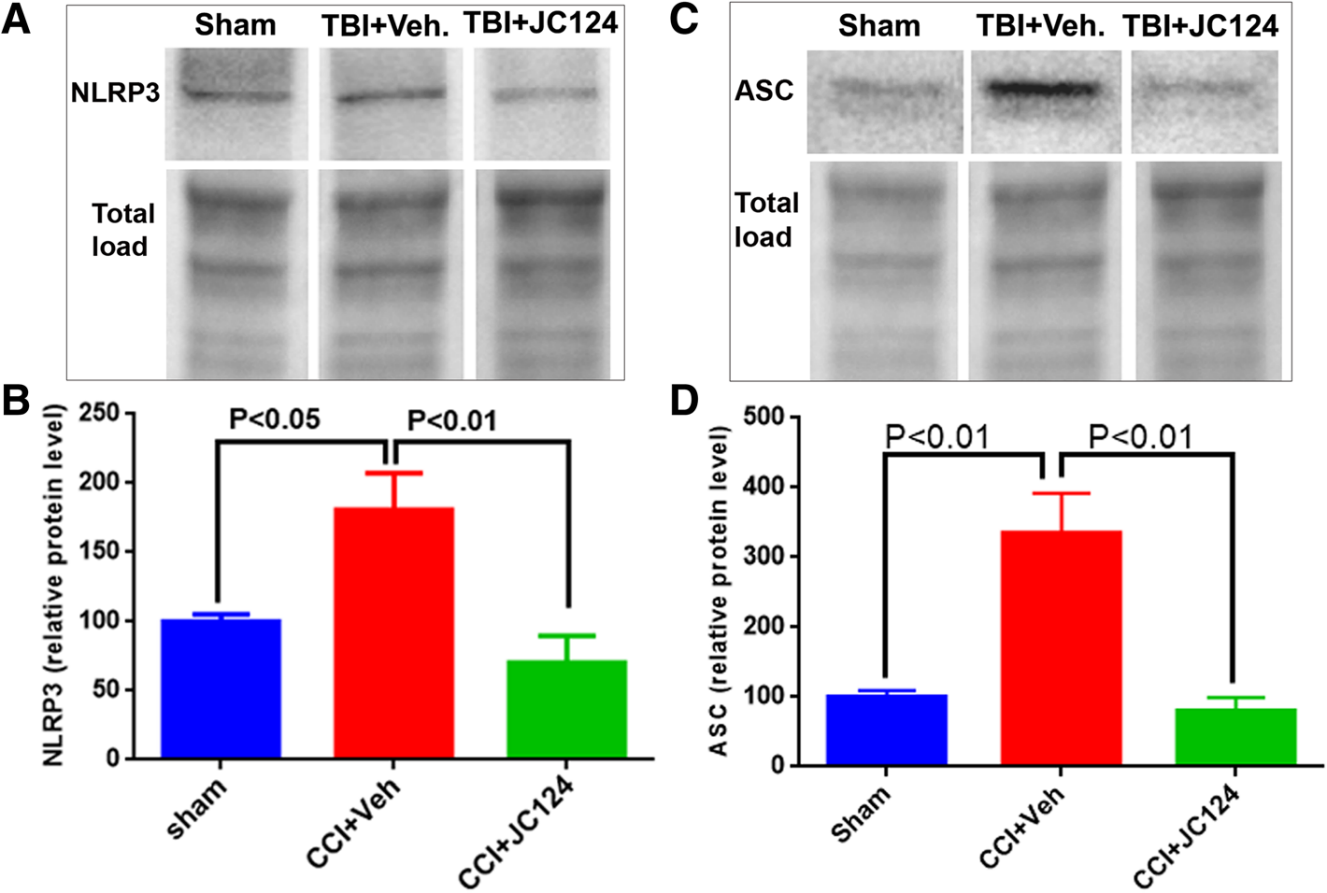
JC124 treatment blocks TBI-induced activation of NLRP3 inflammasome in the injured brain. Representative western blotting images showing the 120 kDa band of NLRP3 (**a**) and 24 kDa band of ASC (**c**) detected in the ipsilateral hippocampal lysate from sham, TBI-vehicle, and TBI-JC124 animals. Quantification analysis showed significantly higher level of NLRP3 (**b**) and ASC (**d**) in the injured vehicle group compared to sham (*p* < 0.05, *p* < 0.01, respectively). In injured JC124-treated group, the injury-induced upregulation of both NLRP3 (**b**) and ASC (**d**) was significantly reduced compared to vehicle group (*p* < 0.01), and the expression level for both proteins was similar to sham

Inflammasomes are essential players in mediating inflammatory response. Recent studies have found that NLRP3 inflammasome is activated in the peri-injury cortex following TBI in a rat weight drop model [13], and a mouse CCI model [26]. Using western blotting analysis, we assessed the protein expression levels of NLRP3 and its adaptor protein ASC in the injured ipsilateral hippocampus in our rat CCI model at 2 days following a moderate injury. The rabbit anti-NLRP3 antibody that we used recognizes a 120 kDa band size, whereas the rabbit anti-ASC antibody recognizes a 24 kDa band size (Fig. 1a, c). Densitometry analysis obtained from the mean value of two independent experiments and normalized with the total protein loaded revealed a significantly enhanced expression level of both NLRP3 and ASC in the injured vehicle group in comparison to sham (Fig. 1b, *p* < 0.05; Fig. 1d, *p* < 0.01). In injured animals which received JC124 treatment, the injury-enhanced expression of both NLRP3 and ASC was completely blocked (Fig. 1 b,d, *p* < 0.01). This data suggests that in agreement with other reported studies, TBI induces activation of NLRP3 inflammasome in the injured brain. Moreover, our novel compound JC124 totally abolishes injury-induced activation of NLRP3 indicating its specificity which further validates our previous reports [17, 18].

### JC124 treatment reduces the number of degenerative neurons, activation of caspase-1 in the injured brain, and the cortical lesion volume following TBI

**Fig. 2:**
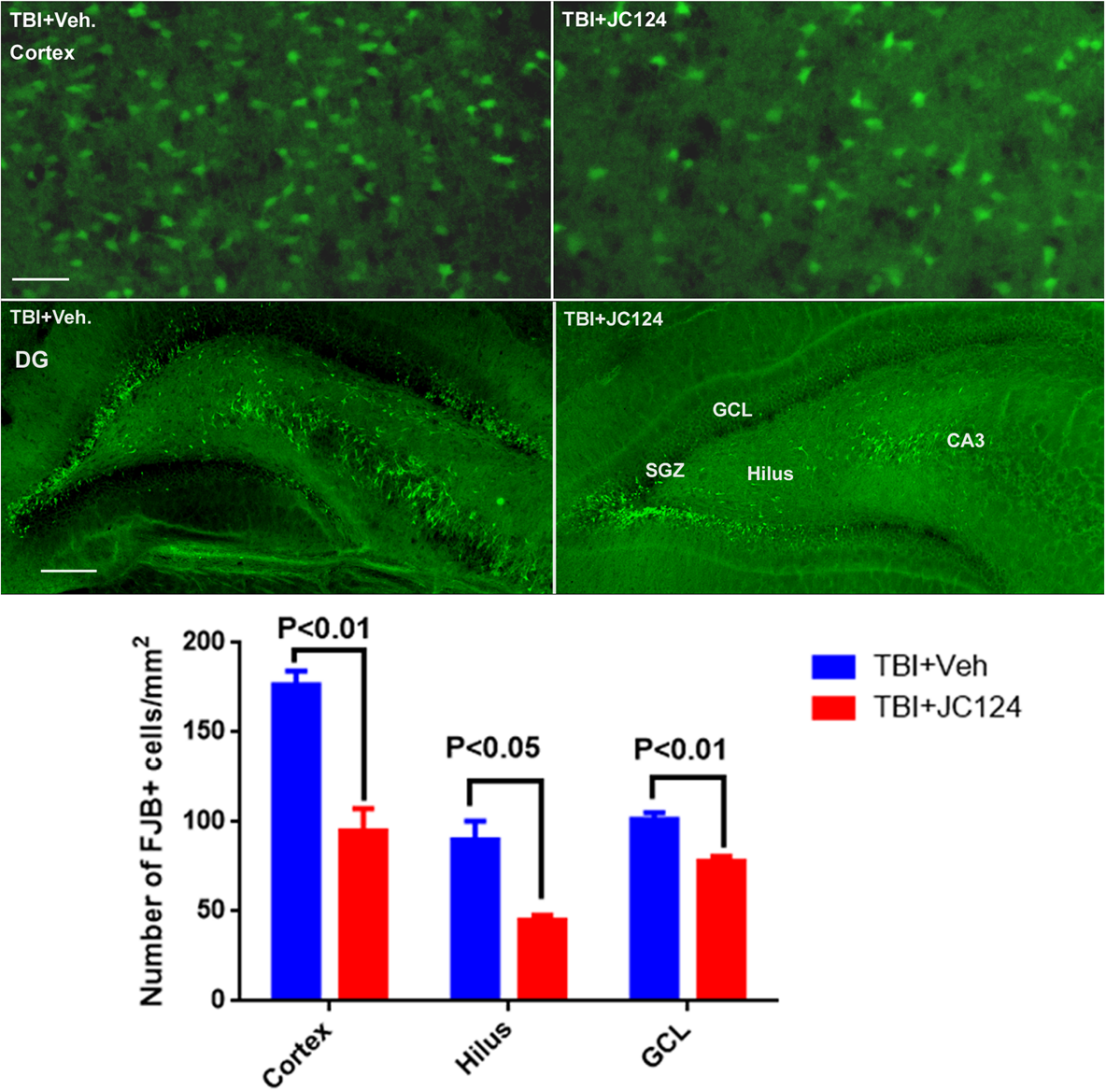
JC124 treatment reduced TBI-induced degenerative neurons. Representative images of ipsilateral cortex and dentate gyrus (DG) taken from FJB stained coronal sections from TBI-vehicle and TBI-JC124 animals. Quantification analysis showed in all three regions assessed including ipsilateral cortex, hilus, and DG granular cell layer, significantly less FJB+ cells were found in JC124-treated group as compared to the vehicle-treated group (*p* < 0.01, *p* < 0.05, and *p* < 0.01, respectively). Bar = 100 μm for cortex, bar = 400 μm for DG

**Fig. 3:**
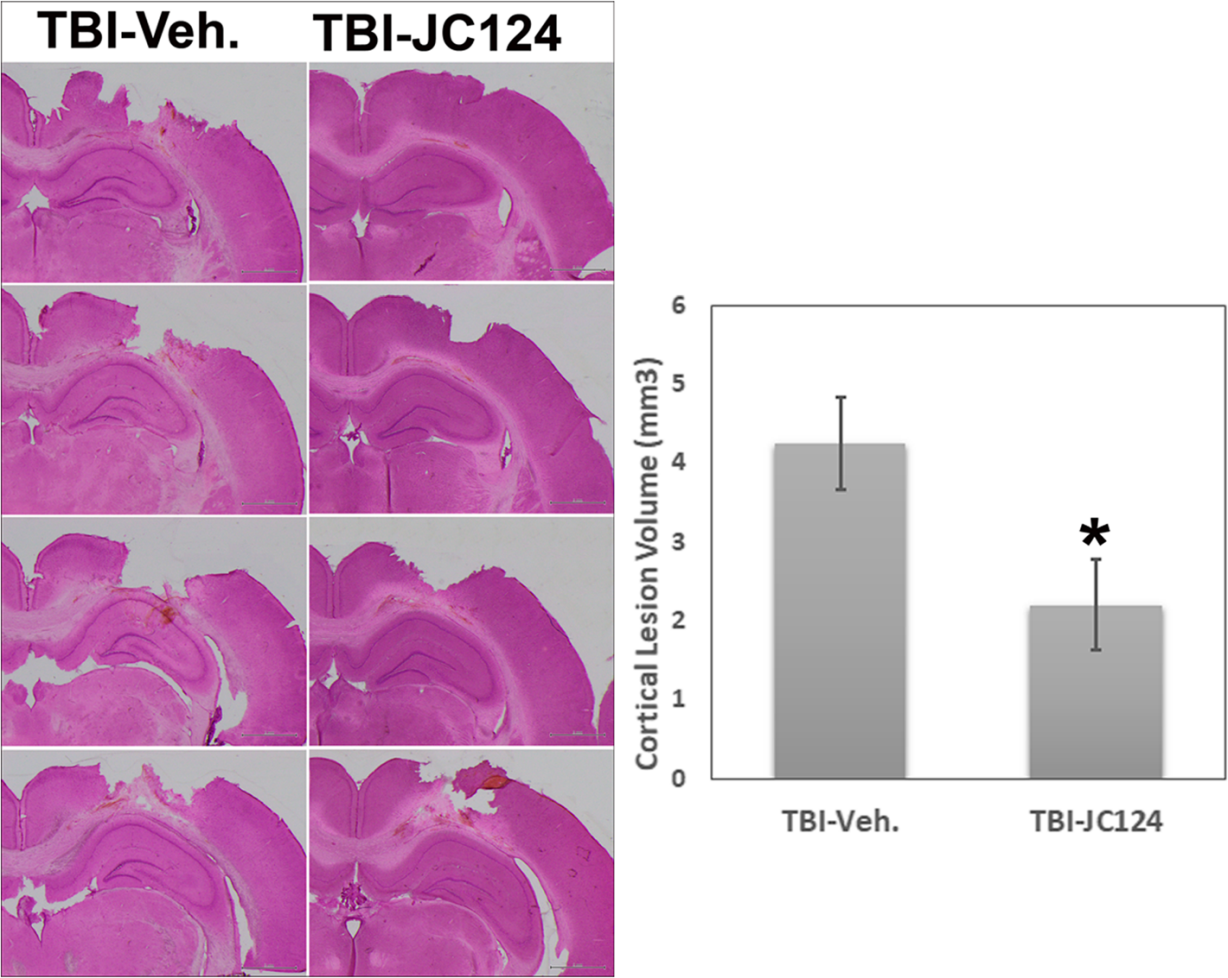
JC124 treatment reduced the cortical tissue damage following CCI. Representative H&E stained coronal brain sections taking from the vehicle and JC124-treated animals showing CCI-induced focal brain tissue damage at 2 days post-injury. Graph showed the quantitative data of lesion volume measurement demonstrating less cortical tissue lose in injured animals treated with JC124 as compared to vehicle-treated animals (*p* < 0.05)

TBI causes significant neuronal degeneration in the region directly underneath the injury impact in areas including cortex, hippocampus, and thalamus, and causes tissue damage. As NLRP3 inflammasome induces activation of caspase-1 and induction of cell death; in this study, we assessed the effect of our NLRP3 inhibitor on neuronal degeneration following TBI using FJB staining. We found that at 2 days following a moderate focal cortical impact injury, extensive FJB+ degenerative neurons were observed in the ipsilateral cortical regions and the hippocampus particularly in the dentate gyrus hilus and granular cell layers (Fig. 2). Compared to the injured vehicle-treated animals, injured animals which received JC124 treatment, the extent of FJB+ cells were significantly reduced in all three regions (Fig. 2). Quantification analysis showed in all three regions assessed including injured ipsilateral cortex, hilus, and DG granular cell layer (GCL), JC124-treated animals had significantly less FJB+ cells as compared to the vehicle-treated groups (Fig. 2, *p* < 0.01, *p* < 0.05 and *p* < 0.01, respectively). No FJB+ cells were found in sham animals. Moderate CCI injury causes a focal tissue damage at the impact site. We found that the cortical tissue damage in injured animals treated with JC124 was significantly less severe compared to injured vehicle-treated animals when lesion volume was assessed (Fig. 3, *p* < 0.05).

**Fig. 4:**
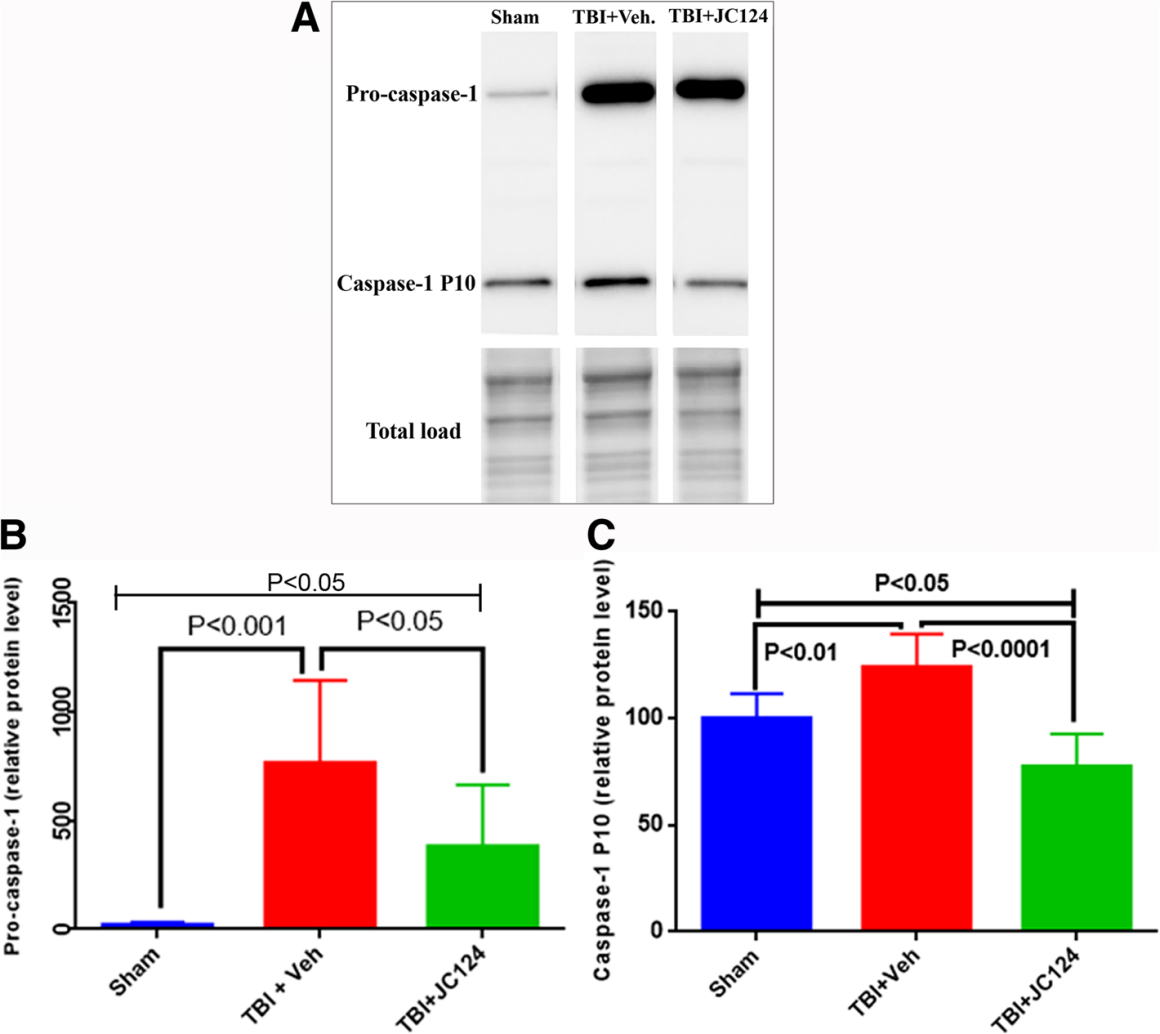
JC124 treatment reduced the post-injury Caspase 1 expression level. **a**) Representative Western blotting image showing the 50kD band of pro-caspase 1 and 10kD band of caspase P10 fragment detected in the ipsilateral hippocampal lysate from sham, TBI-vehicle and TBI-JC124 animals. **b**) Quantification analysis showed significantly higher level of pro-caspase 1 in both injury groups compared to sham and a reduction in JC124 treated group compared to vehicle group (*p* < 0.001 and *p* < 0.05, respectively). **c**) The expression level of caspase 1P10 fragment was significantly higher in the TBI-vehicle group compared to sham and TBI-JC124 group (*p* < 0.01 and *p* < 0.0001, respectively)

To assess caspase-1 activation following TBI, we measured its protein expression levels in the injured hippocampus using western blotting analysis. The caspase-1 antibody we used detects the full-length pro-caspase-1 at 50 kDa and the cleaved active caspase-1 at 10 kDa (caspase-1 p10) (Fig. 4a). We found that the pro-caspase-1 was expressed at a low level in sham animals, the expression level was much higher in the injured animals in both vehicle and JC124-treated groups. Densitometry analysis obtained from the mean value of two independent experiments and normalized with the total protein loaded revealed a significantly increased pro-caspase-1 expression in both injured groups in comparison to sham level (Fig. 4b, *p* < 0.01, TBI + veh. vs sham; *p* < 0.05, TBI + JC124 vs sham). No difference was found in the injured-vehicle-treated group with JC124-treated group. The expression of caspase-1 p10 was observed in all groups including sham and two injury groups. Densitometry analysis showed that compared to sham, caspase-1 p10 was significantly higher in the injured vehicle group (Fig. 4c, *p* < 0.01), whereas the injured JC124-treated group was at lower level (Fig. 4c, *p* < 0.05). The difference between injured vehicle group and JC124 treated group was also different from the vehicle group had significantly higher expression level (Fig. 4c, *p* < 0.01).

### JC124 treatment reduces IL-1β expression both systemically and focally in the brain

**Fig. 5:**
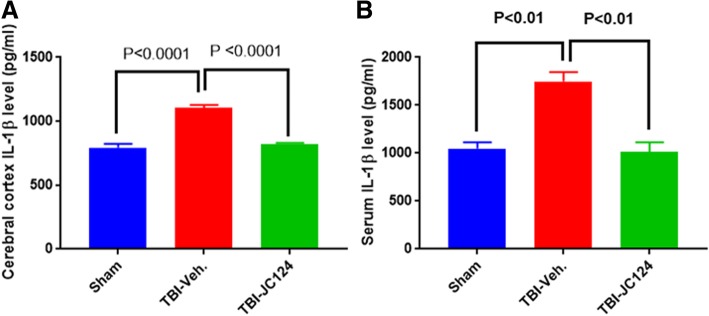
JC124 treatment diminished injury-enhanced focal and systemic expression of IL-1 beta. Expression level of IL-1 beta in the ipsilateral cortex (**a**) and peripheral blood (**b**) was measured with ELISA. In both the ipsilateral cortex (left) and serum samples (right), significantly increased expression of IL-1beta was only observed in the TBI-vehicle group (*p* < 0.01). TBI-JC124 group had similar expression level as sham

**Fig. 6:**
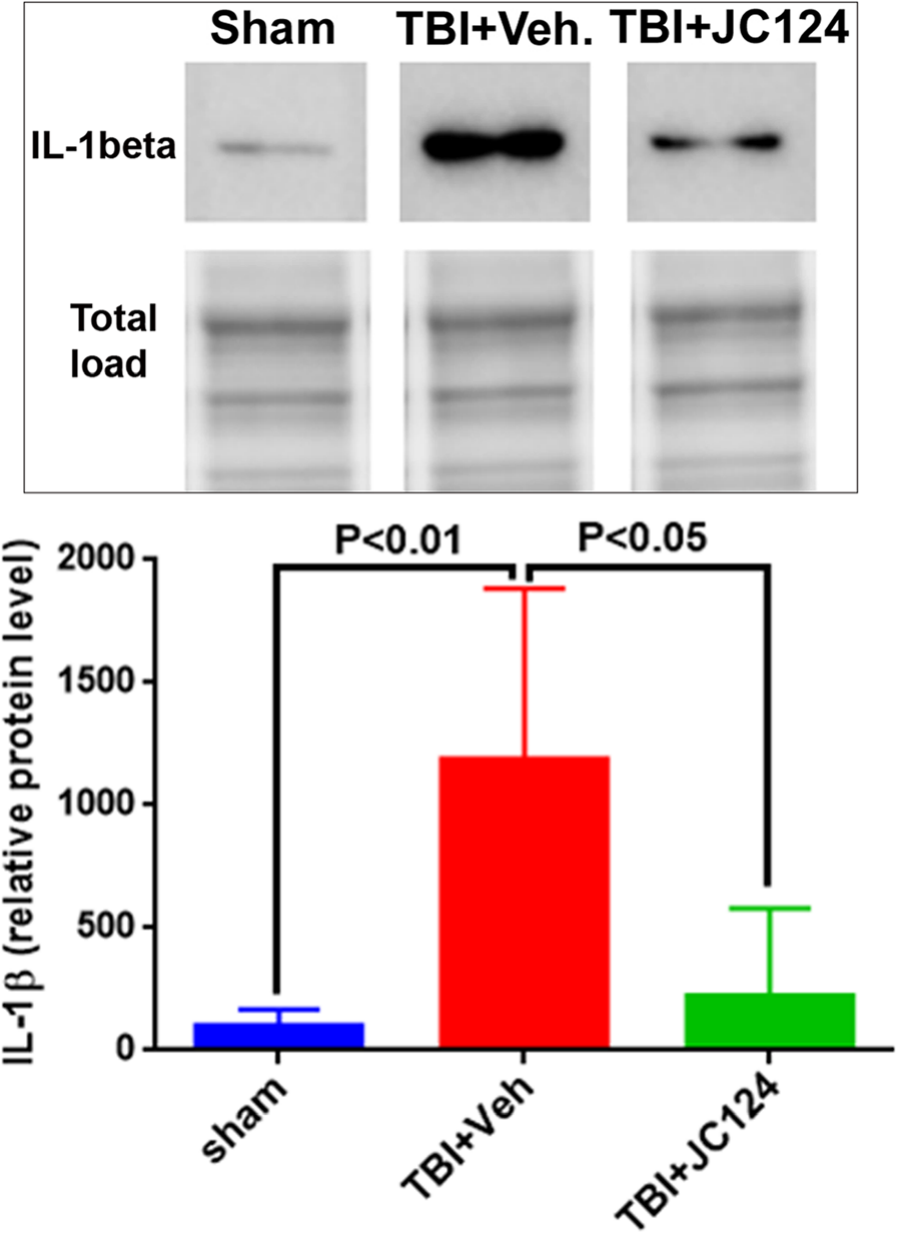
JC124 treatment decreased post-injury expression of IL-1 beta. Representative western blotting image showing the 35 kDa band of IL-1 beta detected in the ipsilateral hippocampal lysate from sham, TBI-vehicle, and TBI-JC124 animals. The densitometry values in the bar graph showed that TBI vehicle group had significantly higher expression of IL-1 beta compared to sham and TBI-JC124 groups (*p* < 0.01 and *p* < 0.05, respectively). Compared to sham, IL-1 beta was slightly higher but not significant in TBI-JC124 group

IL-1β is a pro-inflammatory cytokine, which is the conversion product of caspase-1. Following TBI, increased IL-1β expression in the injured brain at both protein and mRNA levels was universally reported in different TBI models [27]. To examine the specificity and efficacy of our NLRP3 inflammasome inhibitor, using ELISA, we assessed the systemic level of IL-1β in serum samples and focal level in the injured cortex. Using western blotting method, we also measured its expression in the ipsilateral hippocampus. ELISA data showed that at 2 days post-injury, there was a significant increase of IL-1β both systemically in the serum and focally in the cortex in the injured vehicle group compared to the sham group (Fig. 5a, b, *p* < 0.01). Notably, the injured animals treated with JC124 showed significant reduction of IL-1β in both serum and injured cortex compared to the injured vehicle group (Fig. 5a, b, *p* < 0.01), and the expression level was similar to the sham group (Fig. 5a, b). For western blotting, the rabbit IL-1β antibody that we used recognizes a 35 kDa band size (Fig. 6). We found that the expression level of 35 kDa IL-1β was detected in all three groups in the ipsilateral hippocampus, with injured vehicle group showing significantly higher IL-1β expression than the sham and the injured JC124-treated group (Fig. 6, *p* < 0.01 and *p* < 0.05 respectively). No difference was found between the injured JC124-treated group and the sham.

As activation of IL-18 is also triggered by NLRP3 inflammasome, using ELISA we also measured the expression level of IL-18 in serum and in the injured cortex. No significant difference was found between sham and two injured groups in our TBI model (data not shown), similar to what have been reported in other studies [28].

### JC124 treatment reduces inflammatory response

**Fig. 7:**
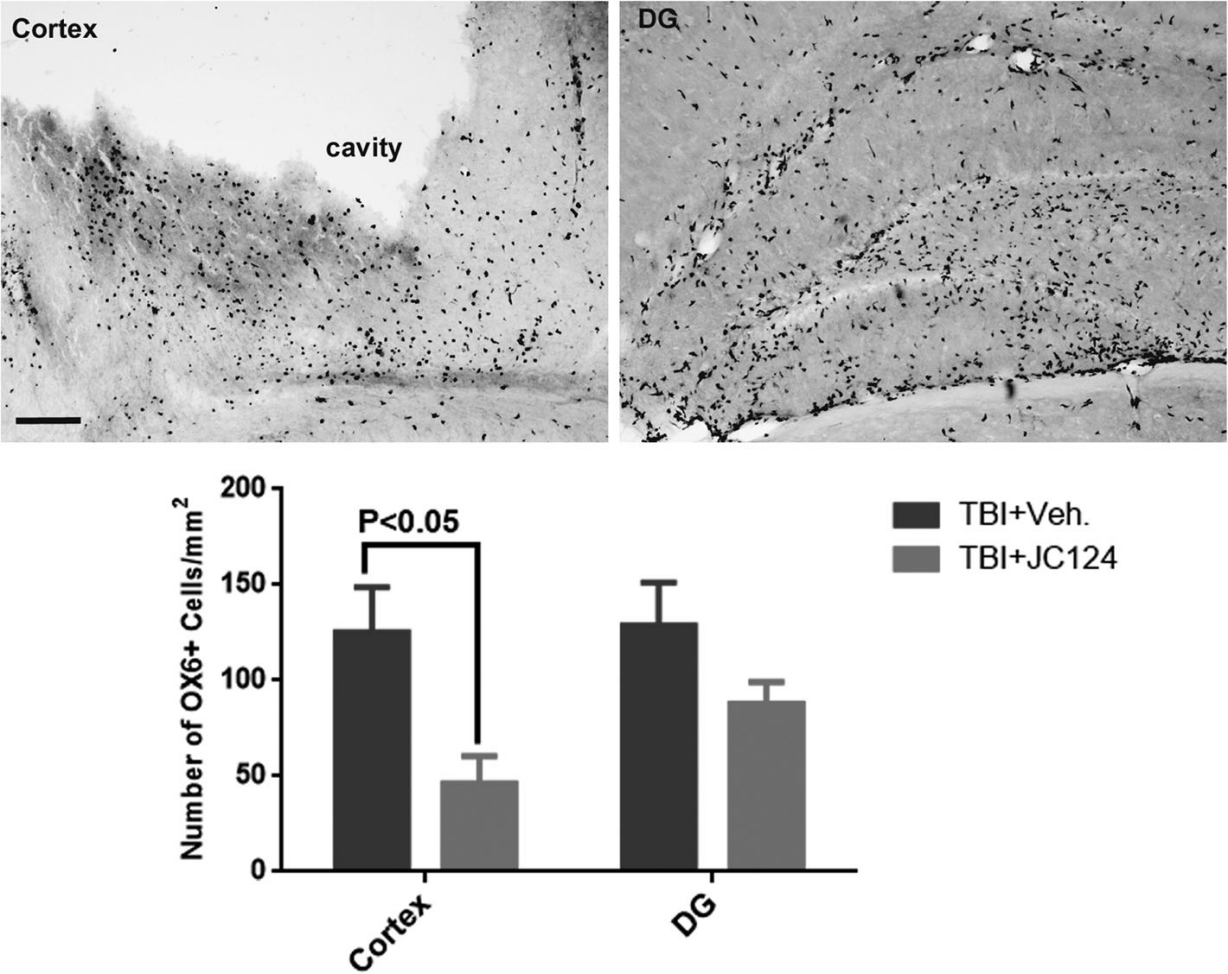
JC124 treatment reduced the number of inflammatory cells. Representative images of OX6+ MHC class II antigen-presenting cells in the injured cortex and the DG. Quantification analysis showed that the number of OX6+ cells was lower in the JC124-treated animals and was significantly reduced in the cortex (*p* < 0.05). Lower number of OX6+ was also found in the DG with a trend towards significance (*p* = 0.06). Bar = 300 μm

NLRP3 inflammasome regulates the production of pro-inflammatory cytokines IL-1β and IL-18 which lead to subsequent inflammatory cascades. To assess the effect of our novel NLRP3 inhibitor JC124 on neuroinflammatory cell response, we used markers, OX6, and ED1 to stain inflammatory cells (infiltrating macrophages and activated microglia). OX6 or ED1 staining was absent in sham animals. In the injured groups at 2 days post-injury, OX6 or ED1 positive cells were apparent in the injured brain in the ipsilateral cortex and hippocampus (Fig. 7). Using ImageJ program, we counted the number of OX6- or ED1-positive cells in the peri-lesion cortex and the dentate gyrus of the hippocampus. Compared to injured vehicle-treated animals, the number of OX6+ cells was significantly reduced in the injured animals received JC124 treatment in the peri-lesion cortex (Fig. 7, *p* < 0.05). A trend of reduction of OX6+ cells was observed in the DG of the hippocampus (Fig. 7, *p* = 0.06). For ED1 staining, no significant difference was found in the number of ED1+ cells between the vehicle and JC124-treated groups in both the cortex and the DG (data not shown).

**Fig. 8:**
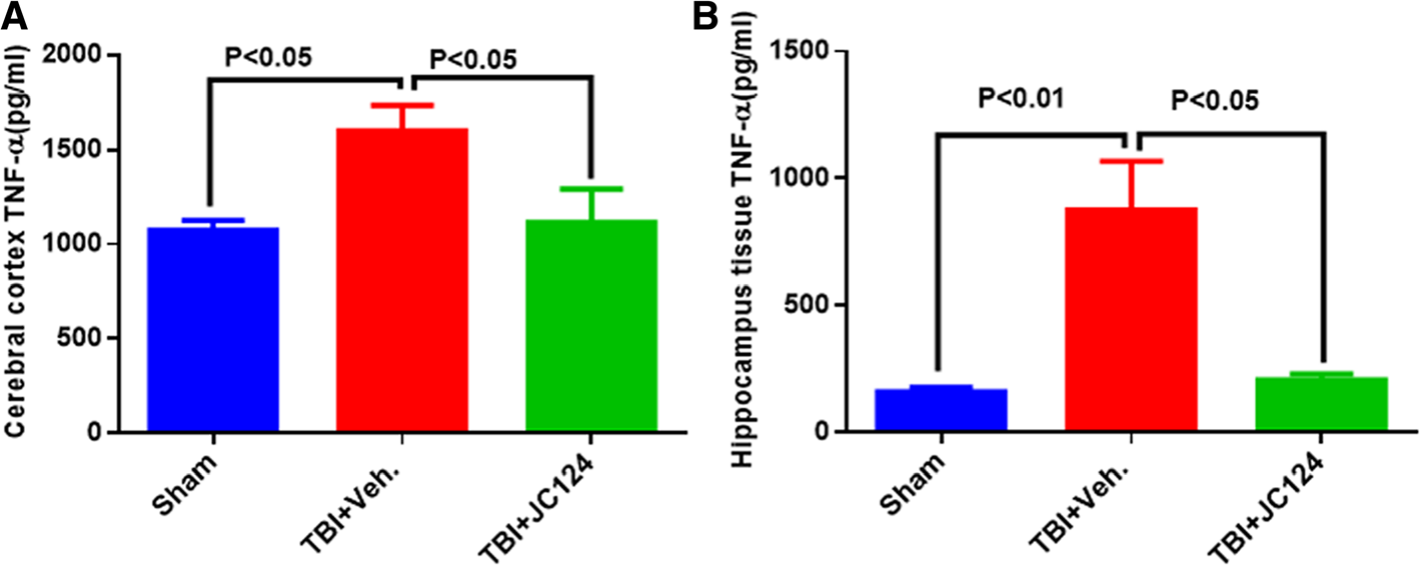
JC124 treatment diminished TBI-elevated inflammatory cytokine TNF-alpha expression in the brain. Expression level of TNF-alpha in the tissue lysate of ipsilateral cortex (**a**) and hippocampus (**b**) was measured with ELISA. In both the ipsilateral cortex (**a**) and hippocampal samples (**b**), significantly increased expression of TNF-alpha was only observed in the TBI-vehicle group (*p* < 0.05 and *p* < 0.01, respectively). Injured JC124 treated group had significantly lower TNF-alpha expression as compared to vehicle treated group (*p* < 0.05), and was not different from sham

**Fig. 9:**
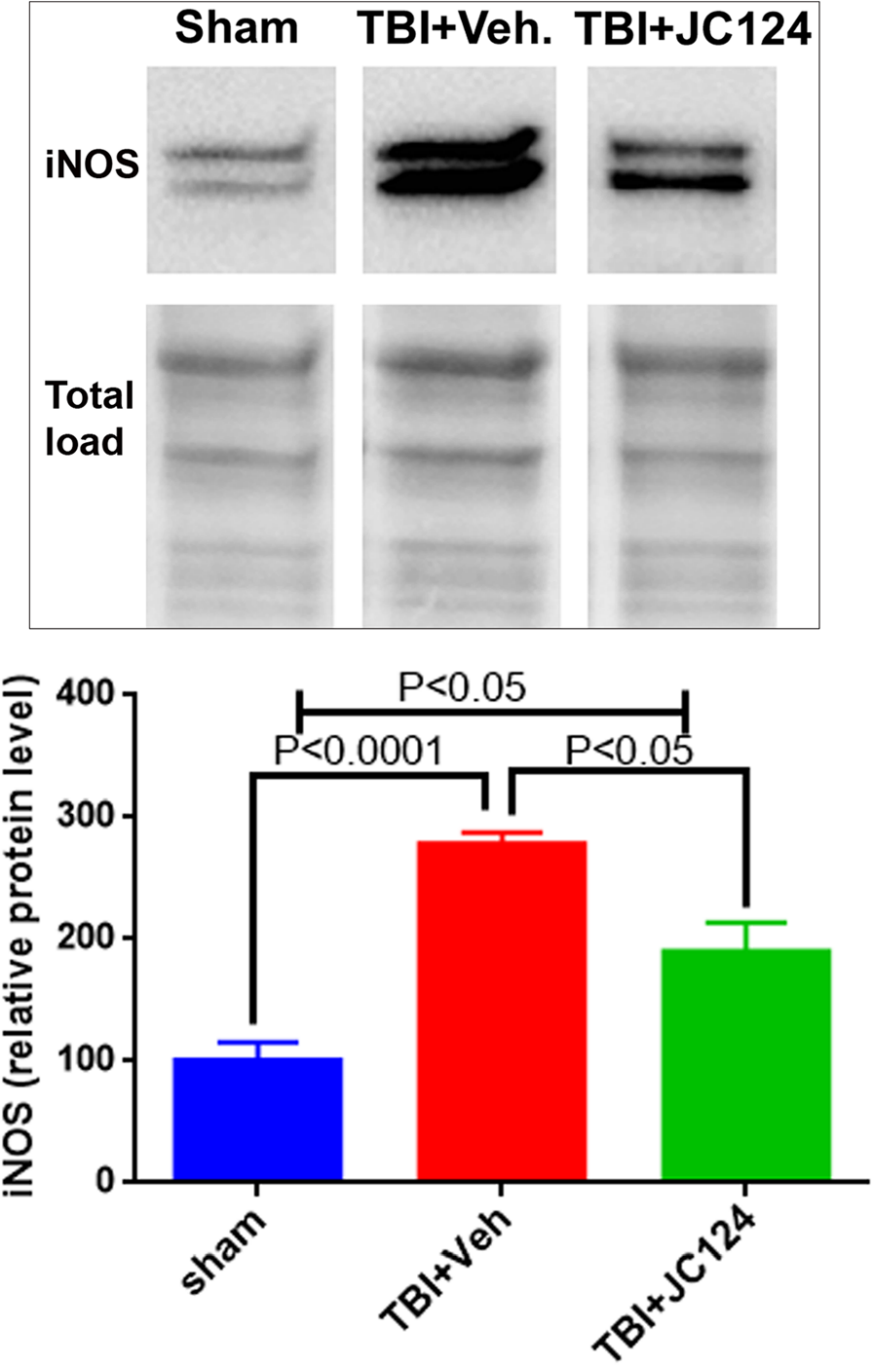
JC124 treatment decreased the expression level of iNOs following injury. Representative western blotting image showing the 130 kDa band of iNOs detected in the ipsilateral hippocampal lysate from sham, TBI-vehicle, and TBI-JC124 animals. The densitometry values in the bar graph showed that TBI induced significantly upregulation of iNOs when compared to sham (*p* < 0.0001), and JC124 treatment reduced the iNOs expression level in the injured animals (*p* < 0.05)

As IL-1β induces an inflammatory response by regulating other inflammatory mediators, to test whether blocking activation of the NLRP3 inflammasome by JC124 treatment can effectively downregulate the downstream inflammatory mediators, we assessed the protein expression levels of TNFα and iNOS. Using ELISA, we measured TNFα protein levels in the ipsilateral cortex and hippocampal lysates. We found that the expression level of TNFα in both the cerebral cortex and hippocampus was increased at 2 days following TBI in the injured vehicle group compared to sham (Fig. 8, *p* < 0.05, *p* < 0.01, respectively), whereas treatment with JC124 significantly downregulated this injury-enhanced TNFα expression in both brain regions to near sham level (Fig. 8, *p* < 0.05). Using western blotting analysis, we assessed protein expression levels of iNOS, a pro-inflammatory M1 phenotype marker and Arginase-1, an anti-inflammatory M2 phenotype marker, in the ipsilateral hippocampus. We found that iNOS protein expression was significantly increased in both injured vehicle and JC124-treated groups as compared to the sham animals (Fig. 9, *p* < 0.01 and *p* < 0.05, respectively). However, injured animals with JC124 treatment had reduced iNOS expression compared to injured vehicle group (Fig. 9, *p* < 0.05). Arginase expression at 2 days post-injury in the ipsilateral hippocampus was slightly higher in the injured vehicle groups compared to sham and JC124 treated, but was not statistically significant (data not shown). This is consistent with published studies showing increased expression of M1 markers at the acute stage following TBI whereas increased M2 markers at a later time point post-injury [20].

## Discussion

Increasing evidence suggest that inflammasomes play key roles in regulating neuroinflammatory response following TBI [29]. The current study has shown that targeting NLRP3 inflammasome with our small molecule inhibitor JC124 is neuroprotective for TBI. Specifically, post-TBI treatment with JC124 at the acute stage following injury significantly decreased injury-induced neuronal degeneration and cortical tissue damage. This protective effect is likely mediated through specific targeting of TBI-induced activation of NLRP3 inflammasome and its downstream neuroinflammatory cascade as demonstrated by completely blocking of TBI-enhanced expression of NLRP3 and its adaptor protein ASC, reduction of downstream caspase-1 activation, reactive oxygen species (iNOS) and pro-inflammatory cytokines IL-1β, TNFα protein expressions. Our results suggest that NLRP3 inflammasome is involved in the development of secondary injury following TBI, and targeting NLRP3 inflammasome is a viable strategy for TBI treatment.

Neuroinflammation is an essential player dictating disease progression in many neurological insults including TBI. Injury-induced neuroinflammatory response, activated by the release of host-derived proteins termed danger-associated molecular patterns, significantly contributes to the progression of secondary injury and impact post-injury recovery. Recent evidence indicates a critical role for the inflammasome complex in initiating neuroinflammatory response after brain trauma [29]. Inflammasomes are essential intracellular multiprotein complexes that direct the innate immune responses to pathogenic stimuli, regulate the activation of caspase-1, production of IL-1β and IL-18, and induction of cell death [7]. Among known inflammasomes, the NOD-like receptors (NLRs) family members NLRP1 and NLRP3 are the most widely studied in the brain and capable to activate caspase-1, IL-1β, and IL-18 [7]. Thus far, activation of NLRP1 and NLRP3 inflammasomes has been reported following TBI in both pre-clinical and clinical studies [30, 31]. In a rat fluid percussive injury model, formation of NLPR1 inflammasome complex, upregulation of caspase-1, and increased IL-1β were detected at 4 h following injury [32]. In a rat weight drop injury model, increased level of NLRP3 and its downstream substrates including caspase-1, ASC, IL-1β, and IL-18 were detected in the peri-injury cortex at both mRNA and protein levels from 6 h to 7 days post-injury [13]. In a mouse cortical impact injury model, increased protein expression of NLRP3, caspase-1, and ASC in the peri-injury cortex was also reported at 1 to 7 days post-injury with the peak expression at 3 days [26]. Study has also reported that TBI led to NLRs and AIM2 inflammasome-mediated pyroptosis in brain microvascular endothelial cells in the injured cerebral cortex in a mouse CCI model [33]. In clinic, NLRP1 and caspase-1 proteins are found in cerebrospinal fluid (CSF) in severe adult TBI patients, and the level is correlated with prognosis [34]. In pediatric patients with severe TBI, increased NLRP3 but not NLRP1 was found in the CSF and was associated with poor prognosis [31]. In a weight drop diffuse injury model using transgenic mice lacking NLRP3, reduced brain tissue damage and inflammatory cell response with preserved cognitive function were observed [35]. In contrast, in transgenic mice which lack NLRP1 or ASC genes, although reduced IL-1β was observed, no protective effect was found following TBI [28]. Collectively, these studies suggest that TBI induces activation of NLRs family members of inflammasomes and activation of inflammasomes particularly the NLRP3 is associated with the injury progression.

Activation of the inflammasome complex is an essential step for the development of neuroinflammation in secondary brain damage. Although the activation pathway is not completely understood, many signals that are related to tissue damage including TBI have been suggested to trigger NLRP3 inflammasome activation including extracellular ATP, K+ efflux, damaged mitochondria, elevated reactive oxygen species, influx of Ca2+, endoplasmic reticulum stress and cathepsin release [36–40]. Among these signals K+ efflux is the best-characterized minimal stimulus for NLRP3 inflammasome activation [41]. Once activated, the NLRP3 inflammasome forms a molecular platform for caspase-1 activation which leads to subsequent release of IL-1β and IL-18 and the eventual amplification of inflammatory responses [7]. The brain is particularly sensitive to IL-1β and IL-18 signaling, as both neurons and glial cells express receptors for these cytokines [42]. IL-1β is the conversion product of caspase-1 activation and triggers NF-KΒ signaling that up-regulates transcription of other pro-inflammatory genes [43]. Ample evidence indicates that IL-1β and IL-18 are involved in the onset and development of the inflammatory cascade following TBI [44–47]. Elevated IL-1β is found in the CSF and brain parenchyma within hours after brain injury in both humans and rodents [44, 48]. It is suggested that the damaging effects of IL-1β is related to its effects on activating other pro-inflammatory cytokines such as TNF-a and IL-6, leading to activation and recruitment of microglia and leukocytes, and disruption of the BBB [42, 48]. Infiltration of macrophages and activation of resident microglial cells further release inflammatory mediators that are cytotoxic to neurons contributing to neurodegeneration and tissue damage [49]. In our study, CCI induces activation of cascape1, leading to upregulated expression of proinflammatory mediators including IL-1β, TNFα, and iNOS, as well as inflammatory cell response in the brain causing eventual neuronal cell degeneration.

Because of the crucial role of NLRP3 inflammasomes in controlling neuroinflammatory response and neural tissue damage following TBI, drug development targeting activation of NLRP3 inflammasome could be a viable therapeutic strategy for TBI. Thus far, studies have reported varying non-specific pharmacological agents with function on NLRP3 inflammasome inhibition having beneficial effect for TBI such as omega-3 fatty acids [50], propofol [51], and resveratrol [52]. Studies also reported that treatment with an anti-ASC neutralizing antibodies can reduce innate immune response and significantly decrease contusion volume in a rat fluid percussive injury model [32], and a NLRP3 inhibitor BAY 11-7-82 post-TBI treatment showing protective effect with reduced brain damage and inflammatory cells was reported [53]. Recently, a small-molecule NLRP3 inhibitor MCC950 has been shown neuroprotective effect in stroke, cerebral hemorrhage, and TBI models [26, 54–56]. In TBI, MCC950 treatment given at 1 and 3 h following a CCI injury in mice, reduction of caspase-1, and IL-1β was observed accompanied with improved motor and sensory function at 1 and 3 days post-injury [56]. When MCC 950 was given i.p. daily for the first 3 days followed by every other day until the end of experiments up to 21 days post-injury, it can attenuate microglia-derived NLRP3 inflammasome activation and production of IL-1β, reduce brain edema, lesion volume, inflammatory cell response, and cell death, as well as improve neurological functions [26].

Our laboratories have recently designed and developed a sulfonamide analog JC124 based on the structure of glyburide. Sulfonylurea-containing compounds such as glyburide and CP-456,773 (now named MCC950) potently inhibit ATP- or hypotonicity-induced IL-1β processing via specific inhibition of the NLRP3 inflammasome [15, 57]. These compounds specifically inhibit the triggering step of NLRP3 activation without affecting the NF-κB signaling-related priming step or the activation of other inflammasomes thus is NLRP3 specific [15, 58]. The mechanism by which these compounds inhibit NLRP3 activation is currently not understood. It is likely that sulfonylurea containing compounds act at downstream of K+ depletion as they do not prevent K+ efflux and the inhibition mechanism is not related to K+ channels. Glyburide was shown to inhibit the ATPase activity of NLRP3, whereas MCC950 does not affect the Ca2+ flux in cells treated with ATP thus with different mechanism [15, 58]. Furthermore, several other small molecule compounds have been reported to target the NLRP3 inflammasome pathway [58–60]. However, the mechanism of action or biological targets of these compounds either act upstream of the inflammasome complex or remain unknown. Glyburide is used in clinic for diabetic treatment, the dose for its NLRP3 inflammasome inhibition effects is at the risk of inducing hypoglycemia, thus cannot be used directly as NLRP3 inhibitor [15]. JC124 was rationally designed based on the structure of glyburide to remove the potential hypoglycemic effects. Our studies have established that JC124 is an active and selective NLRP3 inhibitor by blocking ASC aggregation, activation of caspase-1, and release of IL-1β in macrophages that constitutively express active NLRP3 [16]. Using photoaffinity-labeling probes, we have found that JC124 directly targets inflammasome complex without affecting its ATPase activity, thus representing a novel mechanism (unpublished data). Our studies have also demonstrated the protective effect of this compound in a mouse acute myocardial infarction model [18] and transgenic Alzheimer’s disease models [18, 19]. In this current study, in a rat focal brain injury model, JC124 has shown neuroprotective effects for TBI when given at the acute stage following TBI, similar to what have been reported with MCC950 treatment [56]. Furthermore, compared to reported MCC950 studies done by Xu et al. [26], MCC950 was given during the entire experimental period up to 21 days post-injury, whereas in our study, JC124 was given only during the first 30 h post-injury when NLRP3 inflammasome activation was trigged by brain injury suggesting the benefits of direct inhibition of the inflammasome complex by our novel compound.

Post-traumatic inflammation is detrimental at the early stage but can be beneficial during the chronic stage as it promotes both clearance of debris and regeneration. The target of anti-inflammatory interventions for TBI is to remove danger signals and clear debris during the acute stage, prevent the development of chronic neuroinflammation and promote regenerative immune phenotype in the chronic stage [20]. Thus far, many anti-inflammatory agents have shown beneficial effects in pre-clinical TBI models; however, these effects have failed to translate into clinic. Cautious must be taken in developing new agents targeting neuroinflammation. More studies are needed to evaluate NLRP3 inflammasome inhibitors including our compound JC124.

## Conclusions

TBI triggers activation of NLPR3 inflammasome at the acute stage following injury which plays an important role in propagation of neuroinflammatory cascades in the brain exacerbating secondary tissue damage. We have recently developed a novel small molecular which specifically inhibits activation of NLRP3. In this study, for the first time, we reported that our novel NLRP3 inhibitor is neuroprotective for the injured brain during the acute stage following TBI through specific targeting of NLPR3 inflammasome-triggered neuroinflammatory response. Further studies examining the efficacy of this novel inhibitor during the chronic stage of TBI will provide more information to support its further development and translational value.

## Abbreviations

AD: Alzheimer’s disease
ASC: Apoptotic speck-containing protein
CCI: Controlled cortical impact injury
DG: Dentate gyrus
ELISA: Enzyme-linked immunosorbent assay
IL-18: Interleukin-18
IL-1β: Interleukin-1 β
NLRP3: Nucleotide-binding domain leucine-rich repeats family protein 3
TBI: Traumatic brain injury
TNF-α: Tumor necrosis factor alpha
